# Tumor-associated macrophages: from basic research to clinical application

**DOI:** 10.1186/s13045-017-0430-2

**Published:** 2017-02-28

**Authors:** Li Yang, Yi Zhang

**Affiliations:** 1 Biotherapy Center, The First Affiliated Hospital of Zhengzhou University, No.1 Jianshe East Road, Zhengzhou, 450052 Henan Province China; 2 Cancer Center, The First Affiliated Hospital of Zhengzhou University, No.1 Jianshe East Road, Zhengzhou, 450052 Henan Province China; 3 School of Life Science, Zhengzhou University, No.100 Kexue Road, Zhengzhou, 450001 Henan Province China

**Keywords:** Tumor-associated macrophages (TAMs), Tumor microenvironment, Protumoral activities, Biomarker, Therapeutic target

## Abstract

The fact that various immune cells, including macrophages, can be found in tumor tissues has long been known. With the introduction of concept that macrophages differentiate into a classically or alternatively activated phenotype, the role of tumor-associated macrophages (TAMs) is now beginning to be elucidated. TAMs act as “protumoral macrophages,” contributing to disease progression. TAMs can promote initiation and metastasis of tumor cells, inhibit antitumor immune responses mediated by T cells, and stimulate tumor angiogenesis and subsequently tumor progression. As the relationship between TAMs and malignant tumors becomes clearer, TAMs are beginning to be seen as potential biomarkers for diagnosis and prognosis of cancers, as well as therapeutic targets in these cases. In this review, we will discuss the origin, polarization, and role of TAMs in human malignant tumors, as well as how TAMs can be used as diagnostic and prognostic biomarkers and therapeutic targets of cancer in clinics.

## Background

Non-resolving inflammation in a tumor microenvironment is a hallmark of cancer [1, 2]. Leukocytes, fibroblasts, and vascular endothelial cells together form a tumor microenvironment, with immune cells representing its major component. These immune cells interact with tumor cells to influence the initiation, growth, and metastasis of tumors [3]. Tumor-associated macrophages (TAMs), specifically, are often prominent immune cells that orchestrate various factors in the tumor microenvironment [4, 5].

In general, monocytes/macrophages can be polarized to M1 or M2 macrophages. Classically activated macrophages, also known as M1-polarized macrophages, are activated by cytokines such as interferon-γ, produce pro-inflammatory and immunostimulatory cytokines (e.g., interleukin [IL]-12 and IL-23), and are involved in helper T cell (Th) 1 responses to infection. TAMs are thought to more closely resemble M2-polarized macrophages [6], also known as alternatively activated macrophages, which are activated by Th2 cytokines (e.g., interleukin (IL)-4, IL-10, and IL-13). TAMs play an important role in connecting inflammation with cancer. TAMs can promote proliferation, invasion, and metastasis of tumor cells, stimulate tumor angiogenesis, and inhibit antitumor immune response mediated by T cells, followed by the promotion of tumor progression [6].

With the unraveling of the relationship between TAMs and malignant tumors, TAMs are now being recognized as potential biomarkers for diagnosis and prognosis of cancer, as well as potential therapeutic targets for cancer. In this review, we summarize how TAMs are involved in tumor progression and discuss the clinical significance of TAMs in diagnosis and prognosis of cancers and their use as therapeutic targets in these cases.

## Origins of TAMs

**Fig. 1:**
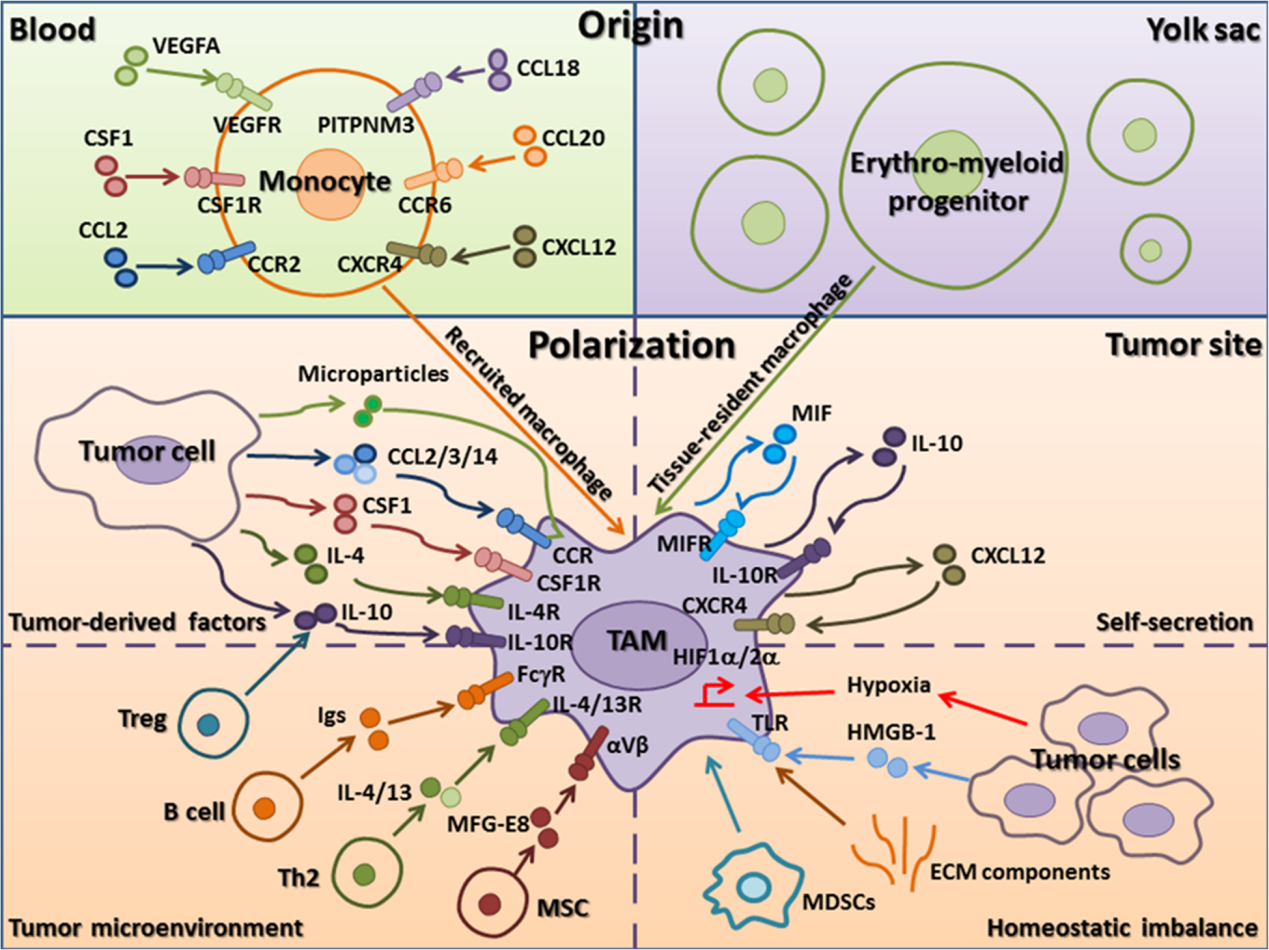
The origin and polarization of TAMs in tumor microenvironments. Recruited macrophages from blood (*green*) and tissue-resident macrophages from the yolk sac (*purple*) coexist in tumors. Recruited macrophages represent the majority of TAMs. Peripheral blood monocytes are recruited locally and differentiate into macrophages in response to various chemokines and growth factors produced by stromal and tumor cells in the tumor microenvironment (CCL2, CSF1, VEGFA, CCL18, CCL20, and CXCL12). Factors that promote the polarization of TAMs to a protumor phenotype can be subdivided into those actively produced by tumor cells (microparticles, CCL2/3/4, CSF1, IL-4, IL-10), those derived from immune system components (Treg-derived IL-10, B cell-derived Igs, Th2-derived IL-4/13, and MSC-derived MFG-E8), those secreted by TAMs (MIF, IL-10, CXCL12), and those resulting from tissue stress (hypoxia, tumor-derived HMGB-1, ECM components) (*orange*). In addition, TAMs can also be differentiated from myeloid-derived suppressor cells in the leukemic stem cell niche

The original understanding of tissue macrophages was that they were solely derived from bone marrow. However, lung alveolar and peritoneal macrophages, Kupffer cells, epidermal Langerhans cells, and brain microglia derived from primitive yolk sac precursors are referred to as tissue-resident macrophages, and they are locally self-maintained. The contribution of locally proliferating macrophages to the pool of TAMs was demonstrated in a Her2/Neu-driven mammary carcinoma animal study [7]. Although there is evidence that all kinds of macrophages can coexist in tumors, recruited macrophages may account for the majority of TAMs and the respective contributions of these macrophages to the various stages of progression in many different tumors cannot be currently quantified. Further studies to characterize TAMs in different human cancers are needed (Fig. 1).

Peripheral blood monocytes from bone marrow are recruited locally and differentiate into TAMs in response to chemokines and growth factors produced by stromal and tumor cells in the tumor microenvironment. Colony-stimulating factor (CSF) 1 is the master regulator and chemotactic factor for most populations of macrophages, whether they are derived from the yolk sac or bone marrow [8]. In a polyoma middle T oncoprotein model, the binding of chemokine (C-C motif) ligand (CCL) 2 to chemokine (C-C motif) receptor (CCR) 2 directly mediated monocyte recruitment to the primary tumor and metastases [9]. In a xenograft model, vascular endothelial growth factor A (VEGFA) recruited monocytes that differentiated into TAMs in the presence of IL-4 and the absence of these TAMs inhibited tumor growth, invasion, proliferation, and angiogenesis [10]. In human breast cancer models, binding of CCL18 to its receptor PITPNM3 mediated the recruitment of macrophages in collaboration with CSF2 [11]. In colon cancer models, macrophage recruitment was mediated by CCL20 binding to its receptor CCR6 [12], the ablation of the chemokine resulted in the loss of monocytes and/or TAMs and inhibition of the malignancy. The accumulation of TAMs in response to CXC chemokine receptor type (CXCR) 4/CXC motif chemokine ligand (CXCL) 12 has been shown to contribute to B16 melanoma progression [13] (Fig. 1).

## Polarization of TAMs

Based on their functions within the tumor microenvironment, TAMs are generally characterized as M2-like macrophages, which express higher levels of anti-inflammatory cytokines, scavenging receptors, angiogenic factors, and proteases than that in M1-type macrophages. These anti-inflammatory cytokines can reprogram the immunosuppressive microenvironment and then promote tumor progression with TAM-derived angiogenic factors, and proteases by multiple ways described in “TAMs promote cancer progression.” TAMs do not become polarized by virtue of their location per se but instead receive signals from the particular microenvironment in which they reside. Currently, a variety of long non-coding RNAs has been demonstrated to impair the function and development of monocyte-macrophages [14]. Moreover, the factors affecting the polarization of TAMs are discussed in detail below (Fig. 1).

### Tumor-derived factors

Several factors produced by tumor cells can reduce macrophage polarization (Fig. 1). Colon cancer cell-derived CSF1 has been shown to drive the recruitment and reeducation of macrophages [15]. Chemokines CCL2, 3, and 14 stimulate macrophage proliferation and polarization in multiple myelomas [16]. IL-10 inhibits the production of pro-inflammatory cytokines and chemokines in macrophages [17]. IL-4 also works in synergy with CSF1 to induce M2-polarized macrophages [18]. Recent evidence indicates that tumor cell-derived microparticles mediate the polarization of TAMs for tumor progression [19]. In addition, prostate cancer-derived cathelicidin-related antimicrobial peptide reeducates macrophages to M2-like phenotype [20]. Hypoxic cancer cell-derived Oncostatin M and Eotaxin differentiate macrophages into M2-polarized phenotype [21]. Soluble MHC I chain-related molecule skews macrophages to immune suppressive alternative phenotype through activation of STAT3 [22].

### Tumor microenvironment

Once monocytes in peripheral blood are recruited to the tumor, the tumor environment rapidly promotes their differentiation into TAMs (Fig. 1). Consistent with the original description of alternative activation, the type 2 cytokine IL-4 secreted from Th2-polarized CD4^+^ cells [23], IL-10 derived from regulatory T cells (Tregs) [24], and immunoglobulin (Ig) from B cells [25] regulate macrophage polarization to the protumor phenotype. IL-13 from Th2 cells may have similar effects on TAM polarization because of overlapping IL-13 and IL-4 signaling cascades that lead to signal transduction and transcription (STAT) 6 activation, although this is yet to be proven in vivo [26]. In addition, mesenchymal stromal cell-derived milk fat globule-epithelial growth factor 8 protein (MFG-E8) [27] has been shown to enhance M2 polarization of macrophages.

### Self-secretion

Recently, migration inhibitory factor (MIF) from macrophages was reported to be an important determinant of TAM polarization in melanoma-bearing mice [28]. MIF deficiency or treatment with an MIF antagonist was shown to attenuate tumor-induced TAM polarization and reduce the expression of proangiogenic genes in TAMs. In addition, tumor-infiltrated macrophages could produce IL-10 to promote TAMs self-polarization [29]. Another study found that autocrine CXCL12 production modulated differentiation of monocytes toward a distinct program with proangiogenic and immunosuppressive functions [30] (Fig. 1).

### Homeostatic imbalance

Hypoxia seems to promote malignant conversion and metastasis, which is mediated primarily through hypoxia-inducible factor (HIF)-1α and HIF-2α. Both of these factors can also regulate macrophage function [31]. The presence of high-mobility group box 1 protein (HMGB1), extracellular ATP, and other normally intracellular molecules is detected by a class of receptors on the surface of macrophages called Toll-like receptors (TLRs). Both TLR2 and TLR6 signaling can promote lung cancer progression by inducing tumor necrosis factor-α (TNF-α) production of macrophages [32]. Tumor-derived extracellular matrix (ECM) components, including biglycan and hyaluronan, are potentially important factors in directing TAM polarization via TLR2 and TLR4 [33]. Crucially, these ECM components do not bind to TLRs in non-inflamed tissue but become TLR ligands following protease cleavage or interaction with reactive oxygen or nitrogen species, thereby forming putative sensory pathways for the detection of inflammation and tissue disruption. In addition, TAMs can also be differentiated from myeloid-derived suppressor cells (MDSCs) in the leukemic stem cell niche [34] (Fig. 1).

## TAMs promote cancer progression

**Fig. 2:**
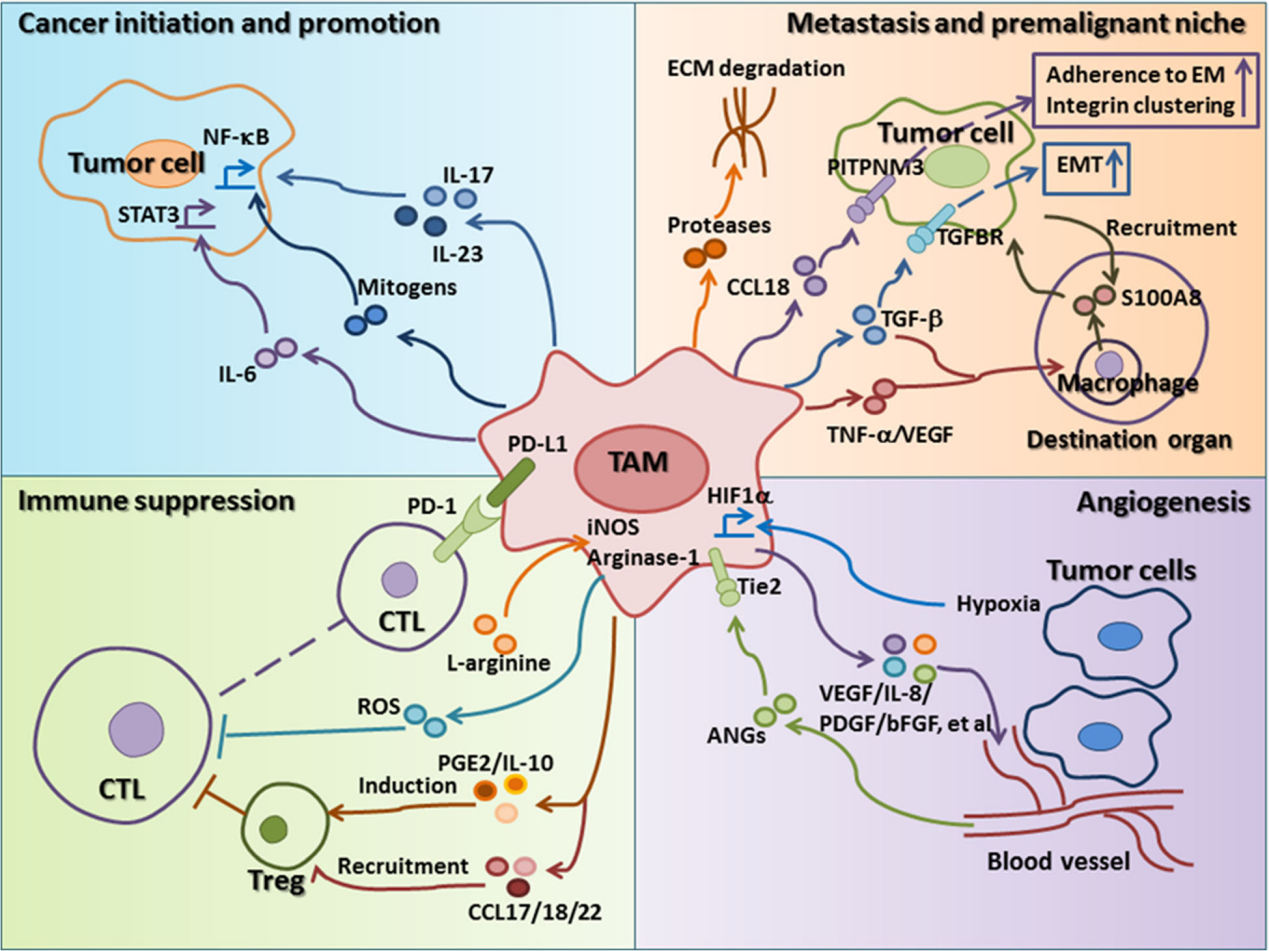
The effects of TAMs on tumor progression. The protumor functions of TAMs include cancer initiation and promotion (*blue*), immune suppression (*green*), metastasis, establishment of a premalignant niche (*orange*), and promotion of angiogenesis (*purple*). (1) TAMs can produce cytokines such as IL-6/IL-17/IL-23 or mitogens to induce the initiation and progression of cancer via the NF-κB or STAT3 signaling pathway in tumor cells. (2) Suppression of CTL proliferation by TAMs is at least partly dependent on metabolism of l-arginine via iNOS or arginase I, which results in ROS production. TAMs inhibit CTL responses via PD1/PD-L1 signaling pathway. TAM-derived PGE2 and IL-10 promote the induction of Tregs, and TAM-derived CCL17/18/22 recruit Tregs, which results in CTL suppression. (3) Neoplastic cell invasion of ectopic tissue can be promoted through protease-dependent ECM remodeling that may directly affect neoplastic migration or the premalignant niche. TAM-derived CCL18 promotes tumor metastasis by triggering integrin clustering and enhancing their adherence to extracellular matrix (EM) in tumor cells. TAM-derived TGF-β plays important roles in initiation and progression of the EMT. TAMs-derived TNF-α, VEGF, and TGF-β can transport through the bloodstream to destination organs, where they induce macrophages to produce S100A8, which further recruits tumor cells to these organs and promotes the formation of metastatic foci. (4) Hypoxia induces HIF-1α expression in TAMs and further regulates the transcription of many genes associated with angiogenesis. Subsets of Tie2^+^ TAMs can interact with mural cells/pericytes to regulate vascular structure

TAMs play particular functional roles in tumor progression, including cancer initiation and promotion, immune suppression, metastasis, establishing a premalignant niche, and angiogenesis. Each of these functions is described below (Fig. 2).

### Cancer initiation and promotion

TAMs connect inflammation and cancer. In 2009, cancer-related inflammation was first defined as a hallmark of cancer. Activated macrophages work in concert with other immune cells in this type of immune response. Evidences suggest that an inflammatory microenvironment promotes genetic instability within developing tumor epithelial cells and infiltrating or resident immune cells such as macrophages in inflammatory microenvironments. Recently, the presence of TAM-derived inflammatory cytokines IL-23 and IL-17 has been shown to be closely associated with cancer progression [35]. Kupffer cells can provide essential mitogens for the promotion of hepatocellular carcinoma through a nuclear factor κB (NF-κB)-dependent signaling mechanism, because its ablation reduced tumor burden [36]. Recent data indicates that TAM-derived IL-6 promotes the occurrence and development of hepatocellular carcinoma via STAT3 signaling [37]. These results suggest that tumor-infiltrated macrophages play an important role in cancer initiation and promotion (Fig. 2).

### Immune suppression

TAMs are the major immunoregulatory cells in tumors, and they participate in inhibiting cytotoxic T lymphocyte (CTL) responses in tumor microenvironments (Fig. 2). In murine tumor models, suppression of CD8^+^ T cell proliferation by TAMs is at least partly dependent on metabolism of l-arginine via inducible nitric oxide synthase (iNOS) or arginase I, which results in the production of reactive oxygen species (ROS) [38]. IL-10 produced by TAMs can induce the expression of costimulatory molecule PD-L1 in monocytes, which can inhibit CTL responses [39]. In addition, TAM-derived prostaglandin E2 (PGE2), IL-10, and indoleamine 2,3-dioxygenase play important roles in the induction of Tregs and TAM-derived CCL17, CCL18, and CCL22 are chemotactic factors for Tregs [40], which results in the suppression of T cells in the tumor microenvironment.

### Metastasis and premalignant niche

The most comprehensively described mechanism by which TAMs promote solid tumor development is to provide factors that enhance metastasis and the establishment of a premalignant niche of malignant cells (Fig. 2).

In human xenograft models, CCL18 is also required for tumor cell invasion and metastasis, playing a role in integrin clustering [41]. Migration on and through the ECM is necessary for tumor cells metastasis, and TAMs are believed to promote tumor cell migration/invasion through the ECM [42]. TAMs can produce proteases, including cathepsin B, matrix metallopeptidase (MMP) 2, MMP7, and MMP9, and cleave the ECM, thereby providing conduits for tumor cells.

The epithelial-mesenchymal transition (EMT) is an important result of the interaction between TAMs and tumor cells. EMT plays a fundamental role in tumor progression and metastasis; therefore, clarifying the regulation of EMT will greatly enhance our understanding of tumor migration and invasion. Accumulating evidence suggests that TAMs play a critical role in the regulation of EMT in cancers. TAM-derived factors play important roles in initiation and progression of the EMT [43].

Also of interest, based on results of studies on animal models, TAMs may play a role in forming premetastatic niches in organs to which the tumor will eventually metastasize. Specifically, TNF-α, VEGF, and transforming growing factor-β (TGF-β), which are derived from TAMs in cancer tissues, are believed to be transported through the bloodstream to destination organs, where they induce macrophages to produce S100A8 and serum amyloid A3. Both S100A8 and serum amyloid A3 can recruit macrophages and tumor cells to these organs and promote the formation of metastatic foci [44]. Thus, TAMs are believed to not only influence their local environments but also to influence macrophages throughout the body and thereby contribute to disease progression.

### Angiogenesis

A few studies have shown that the levels of TAMs are closely associated with the number of vessels in human cancers. Hypoxia is a major driver of tumor angiogenesis. Accumulated macrophages can be found in hypoxic areas of tumor, and particularly in necrotic tissue. HIF-1α, which is expressed in macrophages, regulates the transcription of many genes such as VEGF associated with angiogenesis at hypoxic sites. Genetic analysis has revealed that TAMs can produce VEGF, TNF-α, IL-1β, IL-8 (CXCL8), platelet-derived growth factor (PDGF), basic fibroblast growth factor (bFGF), thymidine phosphorylase, MMPs, and other molecules that are involved in tumor angiogenesis, indicating that TAMs promote the formation of intratumoral blood vessels that provide nutrition for tumor growth [45]. Tie2^+^ TAMs are closely associated with tumor vasculature and have been found crucial for angiogenesis in orthotopic and transgenic tumor models [46], which depend on endothelial cell-produced angiopoietin-2 (ANG2) and Tie2 receptors on TAMs along the vasculature (Fig. 2).

## Diagnostic biomarker of cancer

**Fig. 3:**
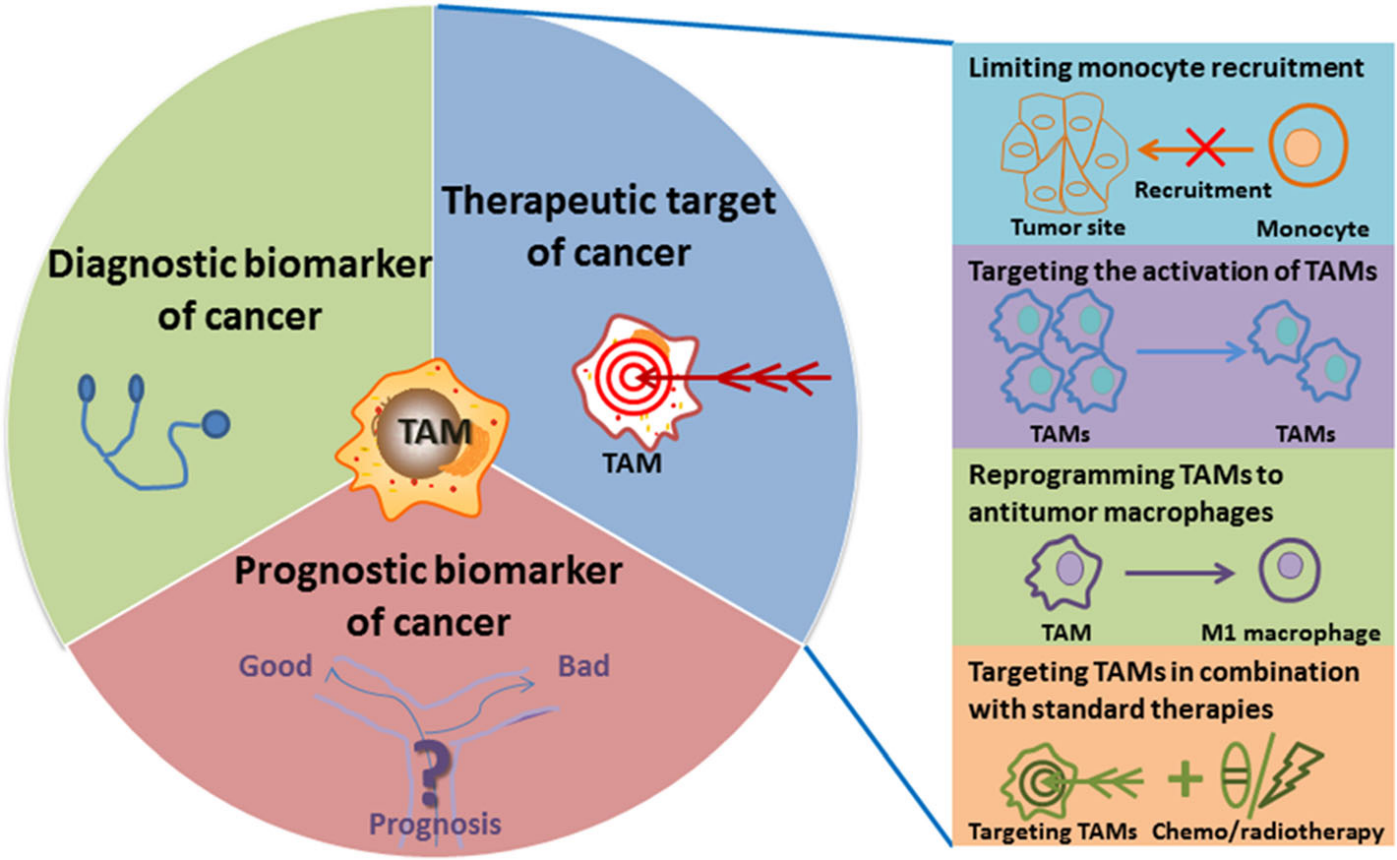
The clinical application of TAMs. As the relationship between TAMs and malignant tumors becomes clearer, TAMs are beginning to be seen as potential biomarkers for diagnosis and prognosis of cancers and as therapeutic targets in cancers. Therapeutic strategies directed at TAMs can be grouped into four areas: limiting monocyte recruitment, targeting the activation of TAMs, reprogramming TAMs to antitumor macrophages, and targeting TAMs in combination with standard therapies

As the relationship between TAMs and malignant tumors becomes clearer, TAMs have begun to be used from bench to bedside, including as potential biomarkers for diagnosis and prognosis of cancer and as therapeutic targets for cancer. First, we will explain how TAMs can be served as potential diagnostic biomarkers of cancer (Fig. 3). Human TAMs are commonly identified by expression of CD163, CD204, or CD206; these biomarkers are not specific for a particular type of cancer.

In our previous study, CD163^+^CD14^+^ macrophages were determined to be potential immune diagnostic markers for malignant pleural effusion (MPE) and have better assay sensitivity than that of cytological analysis [47]. In addition, a serum CD163 value of 1.8 mg/L was set as a cutoff concentration in a survival analysis of patients with multiple myeloma and should be validated in future studies [48]. Tang reviewed the relationship between TAMs and clinicopathological parameters in human breast cancers and addressed the potential value of TAMs as diagnostic biomarkers [49].

Using precision microfilters under low-flow conditions, circulating cancer-associated macrophage-like cells were isolated from the peripheral blood of patients with breast, pancreatic, or prostate cancer. These cells, which are not found in healthy individuals, were found to express epithelial, monocytic, and endothelial protein markers and were observed bound to circulating tumor cells in circulation [50]. These data support the hypothesis that disseminated TAMs can be used as a biomarker of advanced disease, suggesting that TAMs play a participatory role in tumor cell migration.

## Prognostic biomarker of cancer

Due to TAMs’ important role in tumor progression, the level of infiltrated TAMs may be used as a prognostic factor in cancers (Fig. 3). Over 80% of immunohistochemical studies using various human tumor tissues have shown that higher numbers of TAMs are associated with worse clinical prognosis. Recently, we showed that the accumulation of CD163^+^ TAMs in MPE caused by lung cancer was closely correlated with poor prognosis [51]. The results of a study indicate that CD204^+^ TAMs are an independent prognostic factor in esophageal squamous cell carcinoma [52]. A high density of infiltrated TAMs is associated with aggressive features of gastric cancer and is an independent prognostic marker in gastric cancer patients [53]. Macrophage phenotypes (CD68, MAC387, and CLEVER-1/Stabilin-1) provide significant independent prognostic information, particularly in bladder cancers following transurethral resection [54]. Moreover, evidence suggests the expression of inflammation-related genes, especially genes related to polarization of TAMs, contributes to prognosis and is associated with poor clinical outcomes. Therefore, TAMs can be used as a potential biomarker for prognosis of cancers in clinics.

## Therapeutic targets in cancer

As discussed above, there is strong evidence of tumor promotion by TAMs in different cancer models and an increased TAM prevalence correlates with low survival rates in many human cancers. Therefore, targeting TAMs is a novel strategy for the treatment of cancers. Therapeutic strategies directed at TAMs can be grouped into four areas described as below (Fig. 3).

### Limiting monocyte recruitment

One strategy for targeting TAMs is to block monocyte recruitment into tumor tissues. Targeting the CCL2-CCR2 axis is promising due to its important role in monocyte recruitment in tumors. A CCL2-blocking agent (carlumab, CNTO88) has been shown to inhibit the growth of several cancers in animal models. A phase II study of carlumab in metastatic castration-resistant prostate cancer patients showed that this antibody was well tolerated, but that neither blocked the CCL2/CCR2 axis nor showed antitumor activity as a single agent in these metastatic cancer patients [55] (NCT00992186, Table 1). Similar results of Brana et al. showed that carlumab in combination with four chemotherapy regimens for the treatment of patients with solid tumors was well tolerated, although no long-term suppression of serum CCL2 or significant tumor responses were observed [56] (NCT01204996, Table 1). However, according to the results of other study, carlumab was well tolerated, with evidence of transient CCL2 suppression and preliminary antitumor activity [57] (NCT00537368, Table 1).

**Table 1:** Clinical trials of agents that target TAMs for cancer treatment

Action	Agent name	Target	Status	Phase	Tumor type	Effect	Trial number
Limiting monocyte recruitment	Carlumab	CCL2	Completed	II	Metastatic castration-resistant prostate cancer	Well tolerated, no antitumor activity as a single agent	NCT00992186
			Completed	Ib	Solid tumors	Well tolerated, no long-term suppression of serum CCL2, or significant tumor responses	NCT01204996
			Completed	I	Solid tumors	Transient CCL2 suppression, preliminary antitumor activity	NCT00537368
	PF-04136309	CCR2	Completed	Ib	Locally advanced pancreatic cancer	Safe and tolerable, objective tumor response	NCT01413022
	MLN1202	CCR2	Completed	II	Bone metastases	uNTX response rate, 14/43	NCT01015560
Targeting TAM activation	MCS110	CSF1	Recruiting	II	Advanced triple negative breast cancer	NA	NCT02435680
			Recruiting	Ib/II	Advanced malignancies	NA	NCT01643850
			Terminated	I/II	Prostate cancer, bone metastases	NA	NCT00757757
	IMC-CS4	CSF1R	Recruiting	I	Advanced solid tumors	NA	NCT01346358
			Recruiting	I	Advanced, refractory breast or prostate cancer	NA	NCT02265536
	AMG 820	CSF1R	Completed	I	Advanced solid tumors	NA	NCT01444404
			Recruiting	I/II	Pancreatic cancer, colorectal cancer, non-small cell lung cancer	NA	NCT02713529
	PLX7486	CSF1R	Recruiting	I	Advanced solid tumors	NA	NCT01804530
	PLX3397	CSF1R	Completed	II	Recurrent glioblastoma	Well tolerated, no efficacy	NCT01349036
			Completed	II	Relapsed or refractory Hodgkin’s lymphoma	Safe, response rate, 1/20	NCT01217229
			Completed	II	Advanced castration-resistant prostate cancer	NA	NCT01499043
			Recruiting	I/II	Sarcoma, malignant peripheral nerve sheath tumors	NA	NCT02584647
			Recruiting	II	Advanced melanoma, other solid tumors	NA	NCT02452424
			Recruiting	Ib/II	Metastatic breast cancer	NA	NCT01596751
			Recruiting	I/II	Refractory leukemias, solid tumors	NA	NCT02390752
			Recruiting	I	Advanced solid tumors	NA	NCT01525602
	Alemtuzumab	CD52	Terminated	I	Ovarian, fallopian, or primary peritoneal cancers	NA	NCT00637390
			Completed	II	Kidney cancer	NA	NCT00073879
Reprogramming TAMs to antitumor macrophages	ChiLob 7/4	CD40	Completed	I	Advanced malignancies refractory to conventional anticancer treatment	Safe, activate B and NK cells	NCT01561911
	(GM.CD40L) vaccine with CCL21	CD40	Active, not recruiting	I/II	Lung cancer	NA	NCT01433172
	Tremelimumab and CP-870, 893	CD40	Active, not recruiting	I	Metastatic melanoma	NA	NCT01103635
	WP1066	STAT3	Not yet recruiting	I	Recurrent malignant glioma and brain metastases	NA	NCT01904123
	AZD9150 (ISIS-STAT3Rx)	STAT3	Completed	I/Ib	Advanced/metastatic hepatocellular carcinoma	NA	NCT01839604
	β-glucan	MAPK	Completed	II	Stage IV KRAS-mutant colorectal cancer	Compelling, albeit modest, clinical activity	NCT00912327
			Recruiting	I	Neuroblastoma	NA	NCT00911560
			Active, not recruiting	I	Metastatic neuroblastoma	NA	NCT00492167

Sanford et al. demonstrate that a CCR2 antagonist (PF-04136309) can block the mobilization of CCR2^+^ monocytes from bone marrow to tumors in a mouse model of pancreatic cancer and can lead to TAM depletion, causing the inhibition of tumor growth and distant metastasis [58]. PF-04136309, in combination with FOLFIRINOX chemotherapy, was used in a phase Ib trial (NCT01413022, Table 1). This therapy was found safe and tolerable with an objective tumor response [59]. Moreover, the efficiency of the humanized antibody specific for CCR2 (MLN1202) was determined in a clinical investigation (NCT01015560, Table 1).

Treatment with systemic CD11b-neutralizing monoclonal antibodies has been shown to prevent the recruitment of myeloid cells to tumors. It has been shown that the use of Mac-1 (CD11b/CD18) antibodies leads to an improved response to radiation therapy in squamous cell carcinoma xenografts of mice, which is accompanied by reduced infiltration of myeloid cells expressing MMP-9 and S100A8 inside tumors [60].

Because targeting monocytes, prior to being recruited to tumors, has been effective in various cancer models and partial clinical trials, TAMs can be directly targeted as well by other approaches once they invade tumors.

### Targeting the activation of TAMs

TAMs can be targeted at the level of activation using various strategies. CSF1/CSF1 receptor (CSF1R) signaling is critical for the generation of monocyte progenitors in bone marrow and TAM polarization in tumor tissues. For these reasons, CSF1/CSF1R signaling is an attractive target for cancer treatment. Genetic loss of CSF1 results in significantly reduced metastasis and delayed tumor progression in breast and neuroendocrine tumor models [61]. miR-26a expression reduces CSF1 expression in hepatocellular carcinoma [62]. Based on these results, several clinical trials of CSF1/CSF1R inhibitors have been completed or are ongoing (Table 1).

Macrophage surface markers can act as useful therapeutic targets. Mannose receptor CD206 can be exploited as a macrophage-specific target. A single-chain peptide bound to the CD206 receptor was attached to nanobodies that can selectively target CD206^+^ TAMs [63]. Legumain, a stress protein and a member of the asparagine endopeptidase family, can serve as an efficient therapeutic target when overexpressed in TAMs [64]. Targeting surface markers such as scavenger receptor A and CD52 by using immunotoxin-conjugated monoclonal antibodies (mAbs) has been investigated in ovarian cancer [65]. Moreover, the efficiency of alemtuzumab (anti-CD52 antibody) as a tumor treatment in ongoing clinical trials is under investigation (NCT00637390, NCT00073879, Table 1).

Trabectedin (ET743, Yondelis®) was shown to decrease the number of TAMs in tumor tissues by inducing apoptosis of monocytes and macrophages [66, 67]. Based on the favorable results of several phase I, II, and III clinical trials, trabectedin has gained full marketing approval from the European Commission for use in the treatment of ovarian cancer and soft tissue sarcomas and FDA approval in 2015 for use in unresectable or metastatic liposarcoma or leiomyosarcoma [68].

### Reprogramming TAMs to antitumor macrophages

As discussed above, one of the key features of macrophages is their plasticity, which enables them to change their phenotype in the tumor microenvironment. Thus, reprogramming TAMs to an antitumor phenotype is an attractive therapeutic strategy. Antitumor macrophages are good at scavenging and destroying phagocytosed tumor cells [69]. The results of our previous study showed that pseudomonas aeruginosa mannose-sensitive hemagglutinin, which is used in MPE treatment, re-educated CD163^+^ TAMs to M1 macrophages in MPE, suggesting that reprogramming CD163^+^ TAMs can be served as a potential therapeutic strategy of MPE [51].

Nanoparticles are gradually used in polarization of TAMs into antitumor macrophages. Recently, Zanganeh et al. found that ferumoxytol significantly inhibited growth of subcutaneous adenocarcinomas in mice, and this tumor growth inhibition was accompanied by an increase in pro-inflammatory M1 macrophages in tumor tissues [70]. Recent data suggest that bioconjugated manganese dioxide nanoparticles enhance the responses of chemotherapy by inducing TAM toward M1-like phenotype [71]. Synthesized nanoparticles with IL-12 payload can reverse macrophages to antitumor function [72].

CD40 is a surface marker of macrophages that can be used to inhibit cytotoxic functions. The combination of a CD40 agonist with gemcitabine in unresectable pancreatic cancer resulted in regression of tumors by promoting antitumor macrophages [73]. ChiLob 7/4 is an intermediate CD40 agonist and chimeric IgG1, which was also shown to induce pro-inflammatory cytokines, with promising results in CD40-expressing solid tumors and diffuse large B cell lymphoma resistant to conventional therapy in a phase I clinical trial [74] (NCT01561911, Table 1). Other clinical trials of molecules targeting CD40 for cancer treatment are ongoing (NCT01433172, NCT01103635, Table 1).

Activation of the NF-κB pathway also plays an important role in polarization of TAMs to an antitumor phenotype using TLR agonists, anti-CD40 mAbs, and IL-10 mAbs [75]. In addition, regulation of STAT1 activity is an attractive strategy to induce an antitumor phenotype in macrophages because of the increase production of IL-12 in a murine carcinoma model. A small molecule inhibitor of STAT3 (WP1066) was found to reverse immune tolerance in patients with malignant gliomas and to selectively induce the expression of costimulatory molecules CD80, CD86, and IL-12 on peripheral and tumor-infiltrating macrophages [76]. An investigation of this agent to treat recurrent malignant gliomas and brain metastasis is ongoing (NCT01904123, Table 1).

Thymosin-α is an immunomodulating hormone that can reeducate TAMs into dendritic cells, which participate in antitumor host responses and produce high level of pro-inflammatory cytokines. Nanodelivery of thymosin-α is a feasible approach to increase immune activity in cancer patients. Moreover, several clinical trials have confirmed that thymosin-α prolongs survival in patients with metastatic melanomas and advanced non-small cell lung cancers [77].

β-glucan, a yeast-derived polysaccharide, has been shown to differentiate TAMs into an M1 phenotype and is a potent immunomodulator with anticancer properties [78]. The use of β-glucan is currently under investigation in a phase I clinical trial of patients with neuroblastoma [79] (NCT00911560, Table 1). In another clinical trial, a β-glucan polymer (PGG) showed compelling but modest activity in a phase II multi-cancer study [80] (NCT00912327, Table 1). Furthermore, the efficiency of β-glucan is currently under phase I clinical investigation (NCT00492167, Table 1).

### Targeting TAMs in combination with standard therapies

Radiotherapy and chemotherapy are useful treatments in many cancers, and studies have shown that infiltrated myeloid increases after irradiation. However, the interaction between tumor cells and stroma after these therapies remains poorly defined. DNA damage, cell death, and increased hypoxia have been observed in tumors after radiotherapy, which has been shown to lead to macrophage recruitment and promote tumor progression in animal models [81]. Therefore, it is essential to combine TAM targeting with standard therapies for effective tumor treatment.

The HIF-1 pathway is stimulated by radiation-induced tumor hypoxia, and the HIF-1 inhibitor can result in decreased infiltration of myeloid cells into tumors [82]. Even more strikingly, blocking CSF1R signaling appears to enhance the efficacy of several other standard therapies. As such, CSF1R blockade has been shown to increase the efficacy of chemotherapy for pancreatic tumors [83].

## Conclusions

In this review, we discussed the origin, polarization, function, and clinical application of TAMs. TAMs play critical roles in the development and progression of human cancers. Therefore, it will be critical to obtain a better understanding of TAMs to apply clinically, especially as a diagnosis and prognosis marker and a therapeutic target as well. Targeting TAMs is a promising strategy for cancer treatment. Recent ongoing experimental, preclinical, and clinical studies of TAMs have shown encouraging progress. We believe that TAM-targeted therapies will be applied in cancer patients in the future.

## Abbreviations

ANG2: Angiopoietin-2
bFGF: Basic fibroblast growth factor
CCL: Chemokine (C-C motif) ligand
CCR: Chemokine (C-C motif) receptor
CSF: Colony-stimulating factor
CSF1R: CSF1 receptor
CTL: Cytotoxic T lymphocyte
CXCL: CXC motif chemokine ligand
CXCR: CXC chemokine receptor type
ECM: Extracellular matrix
EMT: Epithelial-mesenchymal transition
HIF: Hypoxia-inducible factor
HMGB1: High-mobility group box 1 protein
Ig: Immunoglobulin
IL: Interleukin
iNOS: Inducible nitric oxide synthase
MDSCs: Myeloid-derived suppressor cells
MFG-E8: Milk fat globule-epithelial growth factor 8 protein
MIF: Migration inhibitory factor
MMP: Matrix metallopeptidase
MPE: Malignant pleural effusion
NF-κB: Nuclear factor κB
PDGF: Platelet-derived growth factor
PGE2: Prostaglandin E2
ROS: Reactive oxygen species
STAT: Signal transduction and transcription
TAMs: Tumor-associated macrophages
TGF-β: Transforming growing factor-β
Th: Helper T cell
TLRs: Toll-like receptors
TNF-α: Tumor necrosis factor-α
Tregs: Regulatory T cells
VEGFA: Vascular endothelial growth factor A
